# Proteins pinpoint double strand breaks

**DOI:** 10.7554/eLife.01561

**Published:** 2013-10-29

**Authors:** Michael M Cox

**Affiliations:** Department of Biochemistry, University of Wisconsin-Madison, Madison, United States cox@biochem.wisc.edu

**Keywords:** DNA double-strand break, endogenous DNA damage, GFP, fluorescent-protein fusion, spontaneous DNA break, synthetic biology, *E. coli*, Human, Mouse

## Abstract

Combining green fluorescent protein with a protein that only binds to double strand breaks in DNA allows these breaks—which are an important form of DNA damage—to be detected with high efficiency in living bacteria.

**Related research article** Shee C, Cox BD, Gu F, Luengas EM, Joshi MC, Chiu L-Y, Magnan D, Halliday JA, Frisch RL, Gibson JL, Nehring RB, Do HG, Hernandez M, Li L, Herman C, Hastings PJ, Bates D, Harris RS, Miller KM, Rosenberg SM. 2013. Engineered proteins detect spontaneous DNA breakage in human and bacterial cells. *eLife* **2**:e01222. doi: 10.7554/eLife.01222

**Image** Double strand breaks (arrows) detected in living *E. coli*

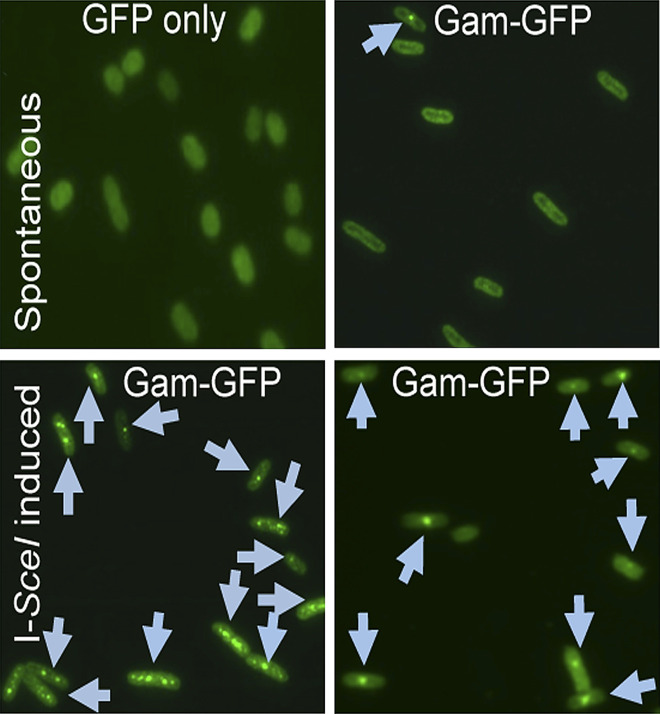

Every genome—whether it is bacterial, archaean or eukaryotic—is subjected to DNA damage on a regular basis. Thousands of DNA lesions can appear per cell per generation in an aerobic bacterial culture, and hundreds of thousands can appear in a single mammalian cell in a day (Hoeijmakers, 2009). Most cells have DNA repair systems to enforce genome stability and, in higher eukaryotes, to prevent cancer. However, these systems can break down (Putnam et al., 2012), and when they do, tumours form. The widespread mutations and rearrangements of chromosomes that are found in tumour cells prevail in what can only be described as genomic chaos (Carter et al., 2012).

Documenting these various genomic insults and their consequences represents a major challenge for medicine, and also for disciplines such as evolutionary biology and cell biology. Now, in *eLife*, Susan Rosenberg of Baylor College of Medicine and colleagues—including Chandan Shee as first author—have provided a promising new tool for the study of one such insult: the double strand break (Shee et al., 2013).

Double strand breaks are considered the most dangerous of all the DNA lesions. If left unrepaired, the resulting chromosome discontinuity often results in death. There are two main ways to repair a double strand break. Recombinational DNA repair is accurate but it relies on the presence of an unbroken homologous chromosome. Non-homologous DNA end-joining, on the other hand, repairs the break, but usually at the expense of adding or deleting genetic information (Chapman et al., 2012; Symington and Gautier, 2011).

Dangerous as they are, double strand breaks are sometimes deliberately introduced into a chromosome. During meiosis, for example, these ‘directed’ double strand breaks are introduced to initiate genetic crossovers between homologous chromosomes (Malkova and Haber, 2012). In yeast, directed double strand breaks are a prelude to an intrachromosomal exchange of genetic information that produces a mating type switch (Haber, 2012). Trypanosomes and other microbial pathogens can evade the immune system by periodically moving new genetic information from a “silent” gene to a highly transcribed locus that encodes a major cellular coat protein; this process is often initiated by a directed double strand break (Deitsch et al., 2009; Vink et al., 2012). Double strand breaks are also central to genetic elements called transposons, and in genomic rearrangements that are integral to the immune system.

Accurate real-time detection of double strand breaks in a cellular genome is thus of great interest in the continuing effort to understand genome maintenance and function. A variety of techniques have been developed to detect and quantify double strand breaks, but they all have one or more deficits in terms of utility, efficiency, sensitivity or specificity. Shee, Rosenberg and colleagues–including co-workers from the University of Texas, the MD Anderson Cancer Center and the University of Minnesota–now report a new approach, based on a protein called Gam, that offers some substantial advantages over existing approaches (Shee et al., 2013).

Gam is encoded by the bacteriophage Mu: this is basically a hybrid of a bacterial virus and a transposon, and it makes a living by moving efficiently within and between bacterial genomes (Baker, 1995; di Fagagna et al., 2003). When an integrated genomic copy of Mu replicates and transposes, the Gam protein protects the free ends of the Mu chromosome as they are transiently exposed. Gam is related to two eukaryotic proteins, Ku70 and Ku80, that are involved in non-homologous DNA end-joining. Whereas the Ku proteins bind to double strand ends, they also interact with an array of other eukaryotic proteins and DNA structures, rendering them less useful for development of a general reagent that binds to double strand breaks. Gam is a simpler system, a single protein with a high affinity for double stranded ends. Rosenberg and colleagues have fused Gam with green fluorescent protein (GFP) to generate GamGFP. When this protein is expressed in a cell, the double strand breaks light up when the cell is illuminated, and this allows the number of breaks to be counted.

In bacteria, GamGFP can detect double strand breaks arising from a variety of sources (Shee et al., 2013). For example, the double strand breaks that occur during DNA replication can be pinpointed (Figure 1), as can the sites where the restriction enzyme Scel cleaves a particular chromosome. Shee *et al*. also provide useful new estimates of the rate of spontaneous break generation. The overall detection efficiency (70–80% in the current study) bodes well for the application of this approach to the detection and quantification of breaks in research into the mechanisms responsible for genome maintenance in bacteria.

**Figure 1.**
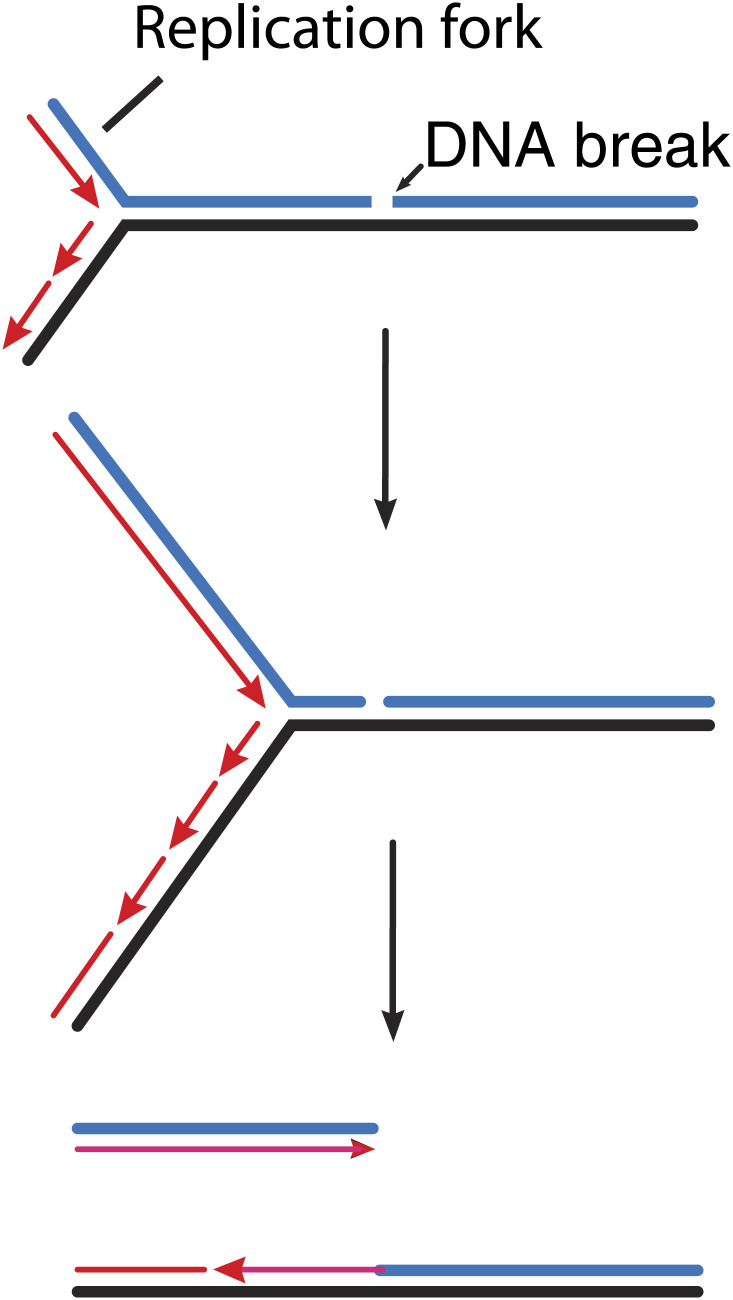
How double strand breaks occur during replication. Many DNA repair processes generate transient single strand breaks in chromosomes. If a replication fork encounters such a break before it is repaired (top and middle), one arm of the replication fork separates to create a double strand break (bottom).

The detection of double strand breaks in the milieu of a eukaryotic chromosome is a much bigger task. Breaks may be buried in chromatin and/or blocked by proteins such as Ku binding to them. In the first round of meiotic cell division, for example, directed double strand breaks are introduced by the protein Spo11, which remains covalently linked to the DNA at the breakage site. Will GamGFP recognize such sites? And in yeast, will GamGFP be able to detect the breaks that are induced to initiate a mating type switch?

Shee et al. demonstrate that GamGFP binds to laser-generated double strand breaks in human HeLa cells, but they find that the recruitment of GamGFP to these sites is inhibited by competition with Ku. Overall, the efficiency of double strand break detection by GamGFP in eukaryotic cells is difficult to assess, and may depend on the source of the double strand breaks that one may want to detect. Nevertheless, this new technology is destined for creative application to important problems in eukaryotic cell biology. The list of potential experiments seems endless.
